# Trauma and Resilience among Latiné Girls: A Mixed Methods Exploration of Positive Adaptations for Trauma and Healing (PATH)

**DOI:** 10.1007/s40653-026-00836-z

**Published:** 2026-03-03

**Authors:** Monica Noriega, Jasmine Ramirez-Miranda, Reid Whaley, David Hoskins

**Affiliations:** 1 Department of Psychiatry and Behavioural Sciences, University of California San Francisco (UCSF), San Francisco, CA USA; 2 Department of Counseling Psychology, University of Oregon, Eugene, OR USA; 3 Department of Population and Public Health Sciences, Keck School of Medicine, Univeristy of Southern California, Los Angeles, CA USA

**Keywords:** Trauma treatment, Adolescent, Latiné mental health, Group therapy, Resilience

## Abstract

The present study measured the feasibility and acceptability of multifamily group therapy in reducing trauma symptomatology and increasing resilience among adolescent Latiné girls. Positive Adaptations for Trauma and Healing (PATH) is a manualized 10-week multifamily group therapy model for Latiné youth in English and Spanish. PATH is a culturally adapted treatment modality that integrates a trauma treatment with mindfulness, positive psychology, and resilience interventions. This study analyzed quantitative and qualitative data collected from 12 adolescent Latiné girls and 16 monolingual Spanish-speaking caregivers (three groups total). Data from pre- and postintervention measures of trauma symptomatology, resilience, and caregiver-related stress were gathered. Postintervention group interviews were transcribed and analyzed using interpretative phenomenological analysis. Latiné girls reported a reduction in the severity of pre- to postintervention posttraumatic stress disorder symptoms (*M*_diff_ = 7.75, *p =* 0.043). Participants also reported feeling more confident at postintervention (*M*_diff_ = 0.73, *p* = 0.05). In addition, caregivers reported a reduction in caregiver stress at postintervention (*M*_diff_ = 6, *p* = 0.043). Three domains were derived from postintervention youth interviews: stronger parent–child relationships, increased self-concept, and increased confidence in the group process. Despite a small sample size, the intervention reduced trauma symptomatology among Latiné adolescent girls in a primary care setting. Group interviews revealed benefits and challenges of multifamily group therapy for Latiné families in urban areas. Future directions for gender- and culture-specific treatment modalities for ethnic minority youth are discussed.

## Introduction

Latinés are one of the largest ethnic minority groups in the United States, with roughly one third of the population under 18 years of age. In 2015, Latiné adolescent girls were more likely to report suicidal ideation and to attempt suicide than their non-Latiné/White peers (Centers for Disease Control and Prevention, 2018). In addition, Latiné adolescent girls are subjected to some of the highest rates of physical, emotional, and sexual violence in the world (Nadal et al., 2017). Simultaneously, Latiné adolescent girls are less likely to engage in mental health treatments due to structural and systemic barriers. Barriers to treatment for this population include lack of bilingual/bicultural clinicians, stigma, mistrust of medical professionals, income barriers, lack of transportation, and language barriers (Goldston et al., 2008; Lê Cook et al., 2013). Gender- and culture-specific treatment modalities for Latiné girls can address this population’s complex and unique needs by facilitating intragroup solidarity. Gender- and culture-specific group therapy modalities in particular may not only reduce symptomatology but also increase connectedness with community for Latiné girls.

## Latiné Female Youth and Need for Culturally Relevant Mental Health Services

Recent studies have shown that that Latiné girls experience higher rates of peer victimization and subsequent depression than non-Latiné peers due to unique factors such as language barriers, perceived immigration status, and physical appearance (Hoskins & Padrón, 2018; Lutrick et al., 2020; Robinson et al., 2021). Research has shown development pathway concerns for girls following trauma and associated posttraumatic stress disorder (PTSD), including increased substance use (D. K. Smith & Saldãna, 2013) and severe psychiatric disorders including internalizing and depressive disorders (Conrad et al., 2014). Studies have shown high rates of psychiatric needs for Latiné girls, in particular, following sexual violence (Basile, 2015). Generational differences for girls have also been found in that first-generation Latinés have shown more severe rates than those born in the United States (Cavanaugh et al., 2013).

Reasons linked to subsequent behavioral health needs for Latiné girls include adversity during the migration experience (Hoskins & Padrón, 2018), family dysfunction (Hoskins et al., 2019; Owen et al., 1998), increased familial responsibilities such as navigating language, and lack of treatment providers with cultural and familial knowledge related to Latiné girls and family (Hoskins & Padrón, 2018). As such, it is likely that the levels of parental stress that caregivers experience related to their own adversity, as well as that of their youth (Hoskins & Padrón, 2018), can contribute to their adolescents’ behavior. Similar to previous research, gender-specific and culturally adapted interventions are needed to disrupt potential and unique pathways for Latiné girls’ behavioral health needs.

### Group Therapy for Latiné Families

Trauma-focused group therapies have been described as useful for reducing feelings of alienation and mistrust of mental health practitioners and health systems (Klein & Schermer, 2000). Aguilera et al. (2018) found that low-income Latiné patients exhibited fewer depression symptoms after participating in a group cognitive behavioral therapy model in a primary care clinic. McDonald et al. (2006) also found high cultural resonance for group-based family therapy in the Latiné community. For Latiné youth, group treatments can be especially efficacious when adapted to incorporate cultural values and community-building activities (Aguilera et al., 2018; Klein & Schermer, 2000). Additionally, caregiver involvement in trauma treatments can reduce internalizing symptoms and increase treatment effectiveness for ethnic minority youth (Hoskins et al., 2017, 2023).

### Positive Adaptations for Trauma and Healing (PATH)

Positive Adaptations for Trauma and Healing (PATH) is a manualized, trauma-focused, 10-week group therapy model for trauma-exposed youth that integrates positive psychology and resilience factors (Hoskins et al., 2017). Hoskins developed the PATH model to address the need for multifamily group therapy for underserved Latiné adolescents and their monolingual Spanish-speaking parents in a primary care setting. The PATH model’s theoretical underpinnings include Kinniburgh et al. (2005) attachment, regulation, and competency model; we also culturally adapted the intervention for the Latiné population and Spanish-speakers (see Hoskins et al., 2023, for adaptation). Specifically, the PATH model incorporates Hinton and Kirmayer’s (2013) and Kinniburgh et al. (2005) emphasis on attunement, emotion regulation techniques, somatic symptoms, and pairing emotion exposure with emotion regulation. PATH facilitators strengthen the parent–child relationship by providing psychoeducation about attunement, praise, routines, and rituals to increase safety and connectedness after a traumatic event (Hoskins et al., 2017). The PATH model also uses interventions from modern resilience and positive psychology theory. For example, participants and caregivers practice noticing positive events, amplifying positive emotions, gratitude, mindfulness, and setting attainable goals.

Hoskins et al. (2017) examined the PATH model’s feasibility in reducing trauma symptomatology among Latiné youth and their Spanish-speaking caregivers. 66% of the youth participating in the preliminary study were girls. Hoskins et al. reported that this was a highly traumatized sample with 56% of participants identifying clinically significant preintervention symptoms. Postintervention analysis showed no participants endorsing clinically significant symptoms of PTSD on the UCLA PTSD Reaction Index (PTSD-RI; Hoskins et al., 2017). Participants in this preliminary study also exhibited increased perceived stress during the posttreatment phase than during the pretreatment phase. Hoskins et al. found that these results aligned with trauma theory such that participants were likely better equipped to acknowledge and tolerate a broader range of emotions after acquiring new skills in the group. The PATH model also increased participants’ capacity to experience and tolerate a broader range of emotions, which could lead to increased resilience (Hoskins et al., 2017).

We tested the PATH model’s feasibility and acceptability with female Latiné adolescents, with the goal of creatin gender- and culture-specific treatments. A mixed methods design was used as part of a growing effort to integrate the voices of marginalized clinical populations into the growing body of literature on evidence-based treatments. We explored whether the PATH model is effective in reducing trauma symptoms and increasing resilience for Latiné girls ages 12–18 years. We hypothesized that participating in the PATH model would result in reductions in trauma symptomatology for adolescent Latiné girls, as measured by the Trauma Symptom Checklist for Children (TSCC) and the PTSD-RI. We also explored whether Latiné girls saw themselves as more resilient after participating in the 10-week PATH model as evidenced by a qualitative analysis of postintervention group interviews.

## Method

### Participants

Inclusion criteria for participation in this study were as follows: (a) experienced three or more forms of traumas such as witnessing or being directly affected by community violence, domestic violence, or immigration; (b) had an impaired caregiving system as identified by the Child and Adolescent Needs and Strengths Screener (CANS); and (c) identified as ethnically Latiné and girls aged 12–18 years. Participants with one or more CANS items with ratings of 2 or higher met the criterion of having an impaired caregiving system. Exclusion criteria included participants who were not able to attend the groups with their caregivers and who did not identify as girls or Latiné. This study was approved by the Human Research Protection Program (Institutional Review Board). All caregivers provided informed consent, and all youth participants provided informed assent.

### Recruitment and Assessment Procedures

Participants were referred by their primary care providers. All physicians worked in a public safety net hospital in the Southwest United States. Service providers referred participants to the PATH model as part of addressing their global health.

The researcher and/or clinical research coordinator met individually with the caregiver and youth up to three times to reduce stigma around mental health services and to determine eligibility to participate in the study. Meetings were facilitated by a bilingual and bicultural psychologist. The families were administered the Trauma History Questionnaire (THQ) and the CANS to determine whether the participant met the inclusion criteria (three or more lifetime traumatic events and an impaired caregiving system). The researcher provided information about the PATH model in Spanish and English, including goals and objectives of the study. Participants and their caregivers were informed of their rights and potential risks of participation in the present study. Once consent was obtained, the researcher (who is bilingual and bicultural) conducted an in-depth intake regarding the family’s needs, strengths, and trauma histories. Efforts to reduce stigma and drop-out rates included exploring and gaining perspectives on the participant’s family history, country of origin, and push and pull factors for immigration.

### Group Structure

The PATH model was facilitated by a psychology doctoral intern and a licensed clinical psychologist. The facilitators conducted three rounds of the PATH model over an 18-month period. Each round consisted of 10 weekly 90-min sessions. Youth and caregivers worked together in a multifamily group therapy setting. Youth and caregivers also met in separate groups in order to learn specific skills and to prepare for parent–child dyadic activities. Additionally, families were given the option to meet individually with the lead clinician at any point during the 10 weeks. The PATH model’s integrity was managed by strict adherence to the interventions as outlined in the manual and weekly supervision with an experienced PATH clinician.

### Intervention

PATH is a manualized multifamily group therapy model that targets trauma symptomatology and the parent–child relationship. Throughout the 10 weeks, participants and caregivers work concurrently to strengthen the parent–child relationship and target trauma symptoms. During Sessions 1–3, caregivers and youth participate in separate groups to learn mindfulness, emotion identification, and emotion regulation skills. These sessions prepare participants for parent–child dyadic activities in a large group. For example, youth and caregivers share gratitude letters and “things I dislike” letters to practice attunement and validation skills during Session 3. In doing so, families can build safety and consistency in the parent–child relationship.

Sessions 3–5 provide psychoeducation about the trauma response and teach grounding and emotion regulation skills. Facilitators and participants develop culturally informed metaphors to explain and externalize aspects of the trauma response, including the *alarma* (trigger), to symbolize the fight/flight/freeze/cling response (see Hoskins et al., 2023, for adaptation content). Participants and caregivers also learn how to identify and regulate somatic manifestations of trauma by building a consistent mindfulness practice.

During Sessions 5–7, caregivers learn the “Communication Tool” in preparation for trauma-focused parent–child dyadic activities. This tool integrates caregiver affect management, consistent attunement, coregulation, and validation skills learned in previous sessions to provide containment for difficult conversations(Blaustein & Kinniburgh, 2010). Simultaneously, youth participants prepare a written trauma narrative and build efficacy in their mindfulness practice. After weeks of preparation, youth share their trauma narrative with their caregiver in the multifamily group during Sessions 7 and 8. Sessions 8–10 target the generalization learned skills across contexts and planning for future goals.

### Measures

Measures of socioemotional functioning, resilience, and traumatic stress were administered the youth and their caregivers before and after participating in the PATH model. All measures were available in English and Spanish, allowing youth and their caregivers to complete them in the language of their choice. The researcher met individually with each family to administer pre- and postintervention measures. The researcher also met with each family within 1 week following completion of the measures to provide feedback regarding changes in symptomatology and to connect the family to mental health resources if needed.

The CANS and the THQ were administered exclusively during the recruitment phase of treatment to determine eligibility for participation. The researcher also determined eligibility using a demographic questionnaire to gather baseline data on participant age, gender, ethnic origins, current living situation, and immigration status. Measures were used to gather data on participants’ pre- and postintervention socioemotional functioning, including the Child Depression Inventory: Short Version (CDI: S), the TSCC, the PTSD-RI, and the Individual Protective Factors Index (IPFI). The researcher also administered the Stress Index for Parents of Adolescents (SIPA) and the Child Behavior Checklist (CBCL) to parents during pre- and posttreatment phases.

### Measures Completed by the Researcher

#### CANS

The CANS was used for recruitment purposes to identify participants who fell into the impaired caregiving symptom category as evidenced by a rating of 2 or higher in the caregiver/family needs and strengths domain. The CANS has been used in past studies with Latiné populations for assessment, treatment planning, and tracking progress in treatment (Hoskins et al., 2017, 2023; Lyons, Chap. 5, 2009). The CANS is also available in Spanish and has norms from U.S. and Latin American samples that increased the relevance of this measure for this sample.

#### THQ

The THQ was also used during recruitment to assess participants’ trauma histories as part of the inclusion criterion of three or more traumatic events. The THQ has 24 items and requires respondents to identify traumatic events by circling yes or no. Respondents who answer yes to experiencing a traumatic event are given space to note the frequency of these events and their age at the time of the event (Green, 1996). The researcher administered the THQ along with the CANS to the youth and their caregivers before beginning treatment.

#### Demographic Questionnaire

The researcher also administered a demographic questionnaire during the pretreatment phase to determine eligibility for inclusion. The demographic questionnaire gathered data from participants and caregivers on the following: age, birth sex, gender, ethnicity, religion/spirituality, years of education, documentation status, number of persons in household, parent country of origin, parent current country of residence, languages spoken, primary language spoken in the home, parent and participant history of deportation, immigration detention (number of days detained, access to health care, access to mental health care), and previous therapy (individual and group).

### Measures Administered to Youth

#### CDI: S

Depressive symptoms were measured using CDI: S. The CDI: S is a 12-item self-report scale for youth aged 7 to 17 years (Kovacs, 2003). The CDI: S asks respondents to select one of three phrases that best represents how they feel (e.g., “I am sad once in a while,” “I am sad many times,” “I am sad all the time”). The CDI: S demonstrated adequate psychometric properties in clinical and nonclinical samples (Cronbach’s α = 0.86; Allgaier et al., 2012).

#### TSCC

The TSCC was used to measure pre- and postintervention trauma symptoms among participants. The TSCC consists of 54 items tailored to measure trauma symptoms through Depression, Posttraumatic Stress, Dissociation, Anxiety, and Anger subscales (Briere, 1996). TSCC items are organized on a 4-point Likert scale ranging from 0 (*never*) to 3 (*almost all the time*). Reliability was deemed adequate for clinical and nonclinical samples with Cronbach’s alphas of 0.85 and 0.66, respectively. Internal consistency was also deemed adequate for clinical and nonclinical samples with Cronbach’s alphas of 0.80 and 0.89, respectively (Briere, 1996).

#### PTSD-RI

The PTSD-RI was used to assess pre- and postintervention PTSD symptoms among participants. This instrument contains 22 self-report items on a Likert scale for children over age 6 years to identify the frequency of their symptoms in the past month (0 = *none* to 4 = *most*; Steinberg et al., 2004). A raw score above 38 suggest that the child is at high risk for developing PTSD or might be currently at criteria for a PTSD diagnosis (Steinberg et al., 2013). The PTSD-RI was found to have adequate reliability of measuring PTSD symptoms as evidenced by pre- and posttest Cronbach’s alphas of 0.85 and 0.88, respectively (Roussos et al., 2005).

#### IPFI

The IPFI is a 71-item self-report instrument that measures resilience across domains of social bonding, personal competence, and social competence (Springer & Phillips, 1997). Items on the IPFI are organized on a 4-point scale (“YES!” “yes,” “no,” “NO!”). The IPFI was normed and validated on over 2,000 youth in a nationwide sample (Springer & Phillips, 1997). This measure has been used to evaluate programs that work with exclusively at-risk populations, including youth with juvenile justice histories (Springer & Phillips, 1997). The IPFI was found to have adequate reliability as evidenced by pre- and posttest Cronbach’s alphas of 0.90 to 0.93, respectively (Springer & Phillips, 1997).

### Measures Administered to Caregivers

#### CBCL

The CBCL is a standardized measure completed by primary caregivers to assess rates of internalizing, externalizing, and behavioral problems among youth ages 6–18 years (Achenbach et al., 2001). Criterion validity is supported by multiple regressions, odds ratios, and discriminant analyses, all at *p* < 0.01 (Nakamura et al., 2009). Nakamura et al. (2009) also found that pre- and posttest Cronbach’s alpha values were 0.97 and 0.96, respectively, indicating that the CBCL has good internal consistency and is a reliable measure of behavioral challenges among children.

#### SIPA

The SIPA was also administered to caregivers before and after this intervention. This assessment is a 112-item self-report questionnaire containing questions regarding issues and stressors faced by parents of adolescents. The SIPA yields several scores on the following: adolescent domain, parent domain, adolescent–parent domain, life stress scale, and total parenting stress. Sheras et al. (1998) found that the SIPA had adequate test–retest validity with an average Cronbach’s alpha of 0.85 and adequate internal consistency with an average Cronbach’s alpha of 0.89.

### Qualitative Measures

The facilitators conducted semistructured group interviews after completion of the PATH model to assess the intervention’s impact on the participants. Youth and caregivers were interviewed in separate groups, and each participant was given an equal opportunity to respond. The facilitator asked four open-ended questions regarding the following: (a) participants’ impressions of the group, (b) how their participation in the group changed the way participants see themselves, (c) whether multifamily group treatment reduced trauma symptomatology, and (d) aspects of the group that participants did not like or found challenging. Responses were recorded and transcribed by the researcher. The researcher identified themes from the transcripts and analyzed them using an interpretative phenomenological analysis framework. This methodology is designed to shed light on the lived experiences of a small number of individuals (J. A. Smith & Osborn, 2015).

### Analyses

The researcher summarized demographic characteristics of the sample using means and standard deviations. Categorical variables such as number of trauma types and ethnicity were measured using frequency counts and percentages. Preintervention to postintervention changes in socioemotional functioning were measured using paired *t* tests.

The researcher and a cofacilitator conducted three rounds of the PATH model, with four families participating in each round. Each youth participant and caregiver consented to participate in the study. A total of 12 Latiné adolescent girls (*N* = 12) aged 12 to 16 years (*M =* 14.3) and 16 monolingual Spanish-speaking caregivers (*N* = 16) participated in the study. One family dropped out of treatment due to Medi-Cal insurance issues.

All participants self-identified as Latiné from various countries, including Mexico, El Salvador, Venezuela, Cuba, and Nicaragua (see Table 1). 50% of participants identified as Mexican or Mexican American, whereas 16% of participants identified as Salvadoran. 58% of participants were U.S. born, and 16% had been in the United States for fewer than 5 years. 58% of participants had at least one caregiver who was undocumented.

**Table 1:** Demographic and clinical characteristics of youth and caregiving system

Characteristic	*n*	%
Sexual orientation/identity		
Heterosexual	10	83
Bisexual	1	8
Pansexual	1	8
Youth time in the United States		
> 1 year	2	16
> 5 years	2	16
Born in United States	7	58
Ethnicity		
Mexican	6	50
Salvadoran	2	16
Nicaraguan	1	8
Mexican/Vietnamese	1	8
Mexican/Cuban	1	8
Venezuelan	1	8
Primary language spoken in the home		
Spanish	6	50
English/Spanish	5	42
English	1	8
Religion/Spirituality		
Catholic	12	100
Christian/Evangelical	0	0
Mothers^a^ (*n = *11)		
Living with youth	11	100
Fathers (*n *= 16)		
Living with youth	6	50
Living in home country	2	16
Living separately from youth in United States	3	25
Caregiver immigration status		
Undocumented	7	58
Citizen	3	25
U visa	1	8
Visa	1	8
Green card	2	16
Other	1	8
Family structure		
Single-parent home	3	25
Extended-family home	5	42
Trauma types	4	1.76
Bullying	8	66
Witnessed domestic violence	8	66
Experienced community violence	11	91
Divorce	6	50
Sexual abuse	3	25
Medical	2	16
Neglect	2	16
Physical abuse	4	33

Mean age 14.2 years

All participants experienced three or more instances of trauma in their lifetime. The mean number of trauma types experienced by each participant was four (*M =* 4, *SD* = 1.76). Participants endorsed histories of bullying, domestic violence, community violence, divorce, neglect, physical abuse, sexual abuse, and medical trauma. 91% of participants had experienced community violence in their lifetime. 66% of participants endorsed witnessing domestic violence between their caregivers in the home, and 50% of participants had parents who were divorced. Notably, 33% reported experiencing physical abuse, and 25% disclosed histories of sexual abuse.

## Results

### Quantitative Results

#### Trauma Symptoms

All PATH participants and caregivers completed pre- and postintervention measures, with the exception of four caregivers who did not complete the SIPA. To examine Hypothesis 1, whether participation in the PATH model would reduce trauma symptomatology among Latiné girls, the researcher analyzed mean pre- to postintervention changes on both the PTSD-RI and the TSCC. At preintervention assessment, youth participants endorsed PTSD symptoms in the at-risk range on the TSCC (see Table 2). At postintervention assessment, participants endorsed a decrease in PTSD symptoms to the normal range on the TSCC, a mean difference from pre- to postintervention scores (*M* = 7.75, 95% CI = 0.29, 15.21) that was statistically significant (*p* = 0.04). Participants’ responses on the PTSD-RI revealed a similar trend in symptom reduction. At preintervention assessment, Latiné girls had more severe rates of PTSD symptoms and in the clinically significant range on the measure. At postintervention assessment, participants’ responses fell within the normal range. Although not statistically significant, the reduction in PTSD symptoms (*M*_diff_ = 9.33, 95% CI = − 4.84, 23.51) from before to after intervention on the PTSD-RI was trending (*p* = 0.18) (Tables 3 and 4).

**Table 2:** Pre- and posttest scores and pared t-Test results

Measure	Pretest M (SD)	Posttest M (SD)	Mean difference (df)	*p* value	95% CI
Youth report					
CDI	60.5	56.58	3.92 (11)	0.28	–3.62, 11.46
IPFI	206.42 (32.81)	216.17 (6.46)	–9.75 (11)	0.3	–29.27, 9.77
TSCC					
Anger	49.33 (11.02)	46.58 (7.57)	2.75 (11)	0.37	–3.66, 9.16
Anxiety	55.08 (14.66)	53.67 (11.03)	1.42 (11)	0.61	–4.52, 7.36
Depression	53.58 (11.82)	47.42 (8.97)	6.17 (11)	0.065	–0.47, 12.81
Dissociation	58.3 (12.16)	51.92 (9.49)	6.42 (11)	0.098	–1.39, 14.22
PTS	56.83 (12.33)	49.08 (8.94)	7.75 (11)	0.043	0.29, 15.21
UCLA PTSD	38.42 (19.87)	29.08 (16.65)	9.33 (11)	0.18	–4.84, 23.51
IPFI	217.7 (28.36)	217.6 (24.94)	0.1 (11)	0.98	
Confidence	17.45 (2.42)	18.18 (2.09)	– 0.78	0.05	
Caregiver report					
CBCL					
Internalizing	64.67 (12.67)	60.83 (11.36)	3.83 (11)	0.11	–1.07, 8.74
Externalizing	57.83 (10.48)	55.17 (12.63)	2.67 (11)	0.28	–2.52, 7.85
Anxious/ Depressed	64.17 (11.48)	61.5(10.01)	2.67 (11)	0.21	–1.74, 7.07
Total	62.42 (13.43)	58.75 (51.25)	3.67 (11)	0.25	–2.97, 10.30
SIPA					
Social alienation	49.87 (14.41)	42.88 (5.84)	5.84 (7)	0.27	–6.80, 20.80
Adolescent domain	50.25 (10.44)	46 (3.30)	4.25 (7)	0.023	0.80, 7.70
Parent domain	47.38 (10.93)	37.76 (4.17)	4.044 (7)	0.043	0.44, 19.56
Total	49.88 (10.51)	43.88 (6.08)	6 (7)	0.043	0.242, 11.76

*CDI* Child Depression Inventory, *IPFI* Individual Protective Factors Index, *TSCC* Trauma Symptom Checklist for Children, *PTS* posttraumatic stress, *UCLA PTSD* UCLA Posttraumatic Stress Disorder Index, *CBCL* Child Behavior Checklist, *SIPA* Stress Index for Parents of Adolescents

**Table 3:** *Youth themes*

Themes	No. of participants endorsing theme (*N* = 12)
Domain 1: Parent–child relationship	
Theme 1: Communication	6
Theme 2: Trust	6
Theme 3: Closeness	7
Domain 2: Self-concept	
Theme 4: Changes in mood	8
Theme 5: Emotion expression	9
Theme 6: Trauma	4
Theme 7: Self-esteem	4
Domain 3: Group process	
Theme 8: Validation	10
Theme 9: Confidence in mental health services	4
Theme 10: Challenges	2

**Table 4:** *Caregiver themes*

Themes	No. of participants endorsing theme (*N* = 16)
Domain 1: Parent–child relationship	
Theme 1: Understanding their adolescent	7
Theme 2: Communication	15
Theme 3: Processing traumatic events	8
Domain 2: Identity as a parent	
Theme 4: “Angry/bad” parent	7
Theme 5: Confidence	7
Theme 6: Minority/acculturation stress	6
Domain 3: Group process	
Theme 7: Normalization	10
Theme 8: Resilience	5
Theme 9: Trust in mental health services	11
Theme 10: Challenges	8

Anger, Anxiety, and Dissociation subscales from the TSCC were also analyzed to measure shifts in pre- to postintervention socioemotional distress. Overall, participants reported modest postintervention reductions in anger, anxiety, and dissociation symptomatology (see Table 2). While none of these shifts were statistically significant, scores on the TSCC’s Dissociation subscale showed a reduction in dissociative symptoms (*M*_diff_ = 6.42, 95% CI 0.29, 15.21). However, this was not statistically significant, but trending (*p* = 0.098).

### Depression Symptoms

Participants reported a reduction in pre- to posttest depressive symptoms (*M*_diff_ = 3.92, 95% CI − 3.62, 11.46) on the CDI: S at postintervention. However, the shift was not large enough to be considered statistically significant (*p* = 0.28). Scores on the TSCC’s Depression subscale also showed a reduction in pre- to postintervention depressive symptoms (*M*_diff_ = 6.17, 95% CI − 0.47, 12.81); this was trending but not statistically significant (*p* = 0.065).

### Resilience

The IPFI was used to measure pre- and postintervention resilience. Results from a paired *t* test revealed a slight increase in participants’ pre- to postintervention self-report of resilience factors (*M*_diff_ = 9.75, 95% CI = − 29.27, 9.77). However, this shift was not statistically significant (*p* = 0.30). Interestingly, there was a pre- to postintervention shift in scores on the IPFI’s Confidence subscale (*M*_diff_ = − 0.78). Specifically, participants rated themselves as more confident in their ability to navigate social and emotional challenges after participating in the PATH model.

(*p* = 0.05).

### Caregiver Report of Youth

Caregivers completed pre- and postintervention CBCL assessments to measure changes in the youths’ socioemotional functioning. Overall, Latiné girls reported a decrease in their total symptom severity per caregiver report (*M*_diff_ *=* 3.67, 95% CI − 2.97, 10.30), which was not statistically significant (*p* = 0.25). Caregivers reported the most symptoms severity on the Internalizing subscale of the CBCL. At preintervention assessment, seven out of 12 youth (58%) exhibited clinically significant internalizing symptomatology, and, at posttest assessment per caregiver report, participants exhibited reductions in internalizing (58% versus 41%). They reported a mean difference on the Internalizing subscale of 3.83 (95% CI = − 1.07, 8.74), which was not statistically significant but trending. Similarly, participants also exhibited reductions on the Anxious/Depressed subscale (*M*_diff_ = 2.67, 95% CI = − 1.74, 7.07). However, this was not statistically significant (*p* = 0.21).

### Caregiver Stress

Caregivers also completed the SIPA. They reported statistically significant reductions in overall stress levels (*M*_diff_ = 6, 95% CI = 0.24, 11.76, *p* = 0.043). Similarly, reductions in levels of stress associated with their adolescents’ behavior and emotional functioning were statistically significant (*M*_diff_ = 4.25, 95% CI = 0.80, 7.70, *p* = 0.023). Reductions in the parent domain also showed a pre- to postintervention decrease (*M*_diff_ = 4.04, 95% CI = 0.44, 19.56), which was also statistically significant (*p* = 0.043). See Table 2.

#### Qualitative Results

After completing 10 weeks of multifamily group therapy, youth and caregivers participated in semistructured group interviews. Three domains and 10 themes emerged from group interviews with youth. The domains were parent–child relationship, self-concept, and group process. Themes in the parent–child relationship domain were communication, trust, and closeness. Four themes were organized under the self-concept domain: changes in mood, emotion expression, trauma, and self-esteem. Finally, the group process domain had the following three themes: validation, confidence in mental health services, and challenges.

Similarly, 10 significant themes emerged from transcripts of group interviews with caregivers. Themes were clustered based on their similarities and common elements and then organized into the following three domains: parent–child relationship, identity as a parent, and group process. Themes under the parent–child relationship domain were understanding their adolescent, communication, and impact of trauma. Similarly, the identity as a parent domain contained three themes: “angry/bad” parent, confidence, and minority/acculturation stress. The final domain, group process, consisted of four themes: normalization, resilience, trust in mental health services, and challenges.

## Discussion

The study results suggest that the PATH model reduced psychiatric symptoms for youth stemming from multiple and various types of trauma as well as caregiver-related stress. The most significant pre- to postintervention changes were decreases in trauma symptomatology, which supports previous studies of the PATH model being effective with Latiné families. This is the first time that PATH was implemented with only Latiné adolescent girls.

The sample for this study included a large percentage of girls who experienced multiple and various types of trauma. Bullying was pervasive in this sample, which has also been noted in emerging literature. Recent studies have shown that that Latiné youth experience higher rates of peer victimization and subsequent depression than non-Latiné peers due to unique factors such as language barriers, perceived immigration status, and physical appearance (Lutrick et al., 2020; Robinson et al., 2021). The current PATH sample also endorsed severe rates of witnessing domestic violence and experienced community violence, which were the most cited traumas for this sample. In addition, this sample of girls experienced caregiver separations in the form of divorce and potential threat of separation via parents being undocumented.

In addition, girls in this sample incurred severe rates of sexual abuse. Sexual violence victimization impacts girls more than boys in the United States, as studies have shown roughly 10.5% of girls versus 4.2% of boys having experienced sexual violence (Johns et al., 2019; Lowry et al., 2017). Parenting style and attachment are protective factors for Latiné girls (Hoskins et al., 2021; Robinson et al., 2024) and are corroborated by the present study’s high rates of attendance and positive outcome results.

For adolescents, studies have reflected an age trend such that depressive symptoms escalate during early adolescence (Kessler et al., 2003; Merikangas et al., 2010). Girls in studies from 2012 to 2018 showed increases in depressive symptoms (Keyes et al., 2019); however, prior to the study by Keyes et al. (2019), a meta-analysis of youth from 1965 to 1996 showed no shift in depressive symptom trends (Costello et al., 2006). And, from 1999 to 2014, suicide rates tripled for girls aged 10 to 14 years (Curtin et al., 2016).

Interestingly, and consistent with the aforementioned research, the highest preintervention scores in the present study were on measures with subscales sensitive to detecting internalizing and depressive symptoms. The present study encompassed two self-report-based perspectives: youth, who filled out the CDI: S and the TSCC, and parents, who filled out the CBCL. All three measures were used to assess pre- and postintervention depressive symptomatology. Of note, all three measures consistently highlighted elevations in preintervention depressive symptoms. At postintervention assessment, reductions in depressive symptomatology were observed in the participants. These findings are consistent with the psychological literature, which has identified a link between trauma exposure in childhood and developing depressive disorders in adolescence (Waldron et al., 2019).

Both the PTSD-RI and the TSCC measured trauma symptoms in the present study. Both measures showed pre- to postintervention decreases in trauma symptomatology; however, only TSCC scores were statistically significant. The PTSD-RI and the TSCC assess PTSD symptoms across domains of intrusion, negative alterations in cognition and mood, avoidance, and physiological arousal. However, the PTSD-RI asks participants to hold a specific traumatic event in mind before answering questions whereas the TSCC does not ask participants to identify a specific experience. It is possible that the discrepancy in outcomes between these two measures in this study reflects this difference in administration.

Participants also endorsed fewer postintervention symptoms on the TSCC’s Anger, Anxiety, and Dissociation subscales. Again, though not statistically significant, these findings suggest that the PATH model could have contributed to these reductions in symptomatology. Similarly, pre- to postintervention changes in youth self-report on the IPFI were not statistically significant. However, the participants reported slight postintervention increases in confidence in social competency on the IPFI. Despite these findings, the results from pre- and postintervention measures of socioemotional functioning trended in the expected direction. As such, this slight increase in postintervention resilience among participants could be linked to improvements in overall social, emotional, and relational functioning.

The quantitative analysis also revealed statistically significant reductions in pre- to postintervention caregiver stress. Family interventions and parental well-being have been heavily documented as important for Latiné families (Hoskins et al., 2023; Meza et al., 2023). At preintervention assessment, caregivers endorsed higher levels of stress under the adolescent domain than under the parent domain. As such, it is likely that caregivers attributed the majority of their stress to their adolescents’ preintervention behavior. Interestingly, there were postintervention reductions in caregiver stress across adolescent and parent domains. These results suggest that caregivers not only experienced reductions in overall stress but also experienced less stress linked to their adolescents’ behavior. Notably, caregivers reported reductions in stress related to their personal challenges following their participation in the PATH model.

Taken together, results from the quantitative analysis of pre- and postassessment measures of socioemotional functioning revealed important findings about the PATH model’s feasibility and acceptability for adolescent Latiné girls and their monolingual Spanish-speaking caregivers. The PATH model contributed to reductions in symptomatology across youth self-report and caregiver report measures of socioemotional functioning. These results suggest that the PATH model likely contributed to reductions in internalizing and externalizing symptomatology. Importantly, the PATH model was successful in reducing trauma symptomatology for Latiné adolescent girls, as measured by the TSCC. Additionally, the PATH model was successful in reducing parental stress for monolingual Spanish-speaking caregivers of Latiné adolescents. Specifically, reductions in pre- to postintervention parental stress were statistically significant. Taken together, these findings provide positive support for the PATH model in working with Latiné adolescents and their families.

### Strengths and Limitations

This study had several limitations, which inevitably impacted the results of the quantitative and qualitative analyses. The sample was small, with only 12 adolescent participants, but adding 16 parents who were involved in the study contributed to the small client sample size. Such a small sample undoubtedly impacted the significance and generalizability of these findings for self-report measures. However, despite the small sample size, findings were trending in the appropriate direction.

Another study limitation was that the same two research administrators facilitated all three rounds of the PATH model. As such, the researcher was unable to determine whether the study results were solely derived from the PATH model and not the unique relationships that the facilitators established with the participants. However, the positive outcomes solely being related to the relationship and/or skills of the two group leaders is unlikely given that this is the third study that has been conducted with different clinicians (Hoskins et al., 2017, 2023). As such, the present study’s results contributed to accumulating evidence supporting the PATH model. Findings could have also been impacted by the results being based solely on participant self-report. Although self-report measures are known to be somewhat biased and unreliable, this study integrated data from multiple sources, including youth self-report and caregiver report measures. In addition, this study supplemented quantitative findings with information from postintervention interviews. As such, the integration of different data sources increased the likelihood of accurate results.

### Suggestions for Future Research

The present study’s findings and limitations highlight many directions for future research in this area. Future studies on the PATH model should feature an all-male participant sample in order to continue to grow the knowledge base and for specific subcultural groups in larger groups as well as the impact of intersectional identities such as race and gender. In addition, future studies should feature larger sample sizes for greater generalizability and clinical applications. The present study collected and analyzed only pre- and postintervention data. As such, future studies featuring longitudinal data on participant functioning several weeks or months after completion of the PATH model could provide valuable information about the intervention’s long-term effects. In the present study, participants exhibited reductions in trauma symptoms immediately after completing the 10-week treatment. However, assessing whether these symptom reductions will remain consistent over a period of time would be important for determining the impact of the intervention. Future research could also assess the impact of PATH facilitators’ cultural identities on treatment outcomes and dropout rates. Continued mixed methods research could also yield important findings about the PATH model’s utility in and outside of a hospital system.

## Conclusions

Latiné adolescent girls continue to exhibit some of the highest rates of trauma exposure in the world. However, Latiné individuals also tend to be the least likely to seek and maintain engagement with mental health services. Under an integrated primary care model, practitioners have the unique opportunity to meet Latiné youth and their caregivers where they are at both culturally and linguistically. With the growing need for culturally appropriate mental health services for this population in the United States, it is imperative for researchers and medical professionals to find solutions by collaborating with marginalized communities.

This study explored the feasibility and acceptability of the PATH model, a manualized, 10-week, multifamily group therapy model for reducing trauma symptoms and increasing resilience among Latiné adolescent girls and their monolingual Spanish-speaking caregivers. Specifically, this study implemented a mixed methods approach by integrating pre- and postintervention measures with postintervention interview data. The PATH model was found efficacious in reducing trauma symptomatology among adolescent Latiné girls. The model was also found effective in reducing stress levels among monolingual Spanish-speaking caregivers. Other clinically significant findings included that the parent–child relationship between adolescent Latiné girls and their monolingual Spanish-speaking caregivers was stronger after participating in the PATH model. Notably, adolescents and their caregivers reported perceiving themselves as more resilient due to improvements in self-concept and self-esteem after participating in the PATH model. Overall, the PATH model was found to be an effective treatment modality to reduce symptomatology, facilitate healing, and create community among Latiné adolescent girls and their families.

## Data Availability

Data can be made available at the request through senior author.
